# Early exposure to thimerosal-containing vaccines and children’s cognitive development. A 9-year prospective birth cohort study in Poland

**DOI:** 10.1007/s00431-014-2412-5

**Published:** 2014-09-05

**Authors:** Dorota Mrozek-Budzyn, Renata Majewska, Agnieszka Kiełtyka

**Affiliations:** Department of Epidemiology, Chair of Epidemiology and Preventive Medicine, Jagiellonian University Medical College, Kopernika 7a St, 31-034 Krakow, Poland

**Keywords:** Ethylmercury, Vaccines, Children, Developmental outcomes

## Abstract

The controversial topic of the early exposure to mercury is regarding ethylmercury, which is present in the thimerosal-containing vaccines (TCVs). The objective of this study was to determine the relationship between the early exposure to TCVs and cognitive development in children during the first 9 years of life. The cohort included 318 children vaccinated in an early period (neonatal and up to 6 months) against hepatitis B and diphtheria-tetanus-pertussis (DTP) using formulation with or without thimerosal. The children’s development was assessed using the Fagan test (6th month of life), the Bayley Scales of Infant Development (BSID)-II (12th–36th month), the Raven test (5th, 8th year), and the Wechsler Intelligence Scale for Children (WISC-R) (6th, 7th, 9th year). Results were determined by multivariable linear and logistic regression, adjusted to potential confounders. Children exposed and not exposed to TCVs in the neonatal period had similar outcomes of cognitive-developmental tests; only the results of BSID-II at the 36th month and WISC-R at the 9th year were significantly higher for those exposed to TCVs. Developmental test results in children exposed to TCVs up to the 6th month of life also did not depend on thimerosal dose.

*Conclusion*: TCV administration in early infancy did not affect children’s cognitive development.

## Introduction

High doses of mercuric compounds are nephrotoxic and neurotoxic. Cumulative exposure to an organic mercury-containing compound, methylmercury, can also produce neurologic and renal damage. It has a long half-life and can cross the blood-brain barrier, where it accumulates and is converted to inorganic mercury [16]. Ethylmercury, a related organic mercury compound, is a constituent of thimerosal, an antibacterial agent used in certain non-live vaccines. Ethylmercury has a much shorter half-life than methylmercury, being rapidly excreted via stools after parenteral administration, but its influence on children’s health has not been determined yet [18, 19]. Nevertheless, the guidelines to limit cumulative methylmercury exposure have been translated to ethylmercury.

The discussion on the safety of thimerosal-containing vaccines (TCVs) started in 1999 (Joint Statement issued by the American Academy of Pediatrics and the US Public Health Service in July 1999) [11]. It was suspected that ethylmercury contained in the vaccines had a harmful effect on children’s neurodevelopment. In fact, the low-dose human exposure to ethylmercury has not been assessed sufficiently, although it is assumed to have a similar effect as the exposure to methylmercury that demonstrated harmful consequences [6, 7, 9]. Since 2004, no vaccines recommended and routinely used in the USA and the European Union to protect preschool children have contained thimerosal [4, 20]. Nevertheless, most countries continue to use TCVs in their childhood immunization schedules.

The objective of this study was to determine the relationship between the early exposure to TCVs and cognitive development in children up to the 9th year of life.

## Material and methods

This is a prospective cohort study, combining environmental monitoring and molecular approaches with comprehensive neurodevelopment assessments. In the analysis, we used data on the vulnerability of a fetus and child to environmental factors, coming from an earlier established birth cohort of children in Krakow, being part of an ongoing, collaborative study together with Columbia University in New York. The study has received the approval of the Jagiellonian University Ethical Committee (Krakow, Poland).

The enrolment (November 3, 2000–August 22, 2003) included only non-smoking women, aged 18–35 years, with singleton pregnancy without illicit drug use and HIV infection, free from chronic diseases such as diabetes or hypertension, and residing in Krakow for at least 1 year prior to the pregnancy. The infants were followed up to the 9th year of life. Each year, mothers were asked to provide information on infants’ health and household characteristics by trained interviewers, who carried out detailed, face-to-face standardized interviews. The Test of Nonverbal Intelligence, Third Edition (*TONI-3*) was administered to mothers. We have included this instrument to adjust to the maternal contribution to the child’s cognitive development.

### Vaccination data

The data on infants’ vaccination history (date of vaccination and type of vaccine) were extracted from the physician’s records. Neonatal thimerosal exposure status was based on hepatitis B vaccination in the first 29 days of life (first dose). A further level of TCV exposure took into consideration hepatitis B as well as diphtheria-tetanus-pertussis (DTPw or DTPa) vaccines up to the 6th month of life.

### Biological samples and analysis

Heavy metal (mercury, lead) concentrations were examined in cord blood (at delivery) and capillary blood (5-year-old children). Whole blood lead concentrations were determined using inductively coupled plasma mass spectrometry CLIA’88 method “Blood lead cadmium mercury ICPMS_ITB001A.” This multielement analytical technique is based on quadruple ICP-MS technology [3]. Mercury levels were measured at the Center for Disease Control by Zeeman graphite furnace atomic absorption spectrometry, using a phosphate/Triton X-100/nitric acid matrix modifier. Cold vapor atomic spectrometry following chemical reduction of mercury compounds was used to measure total mercury in whole blood. More details on blood sample collection and analysis were presented in the earlier publications [9, 10].

### Infant neurodevelopment testing

The Fagan Test of Infant Intelligence (FTII) was conducted at the 6th month of life. The Bayley Scales of Infant Development, Second Edition (BSID-II) was administered at the 12th, 24th, and 36th month of life. The Mental Scale of BSID_II test includes items that assess memory, habituation, problem solving, early number concepts, generalization, classification, vocalization, language, and social skills [2]. Test scores are adjusted to the child’s age to obtain the Mental Development Index (MDI). Test results are in one of four categories: (1) accelerated performance (score >115), (2) within normal limits (score 85 to 114), (3) mildly delayed performance (score 70 to 84), and (4) significantly delayed (score <69). Results <85 were considered as poorer cognitive development.

The test of Raven’s Colored Progressive Matrices (Raven) was administered twice, at the 5th and 8th year of life. The outcomes of the test were measured in terms of centiles. Because the results of this test were generally high, the cut point of poor result category was 75th percentile, which means middle intelligence outcomes.

The Wechsler Intelligence Scale for Children-Revised (WISC-R) was administered at the 6th, 7th, and 9th year of life and generated verbal, non-verbal, and total IQ for the evaluated children. The outcome’s range is from 40 to 160. The category with IQ <110 was considered as the poorer outcomes.

All neurodevelopment tests were conducted at the Department of Epidemiology and Preventive Medicine by carefully trained examiners who were unaware of the child’s exposure. The Bayley Scales as well as the Raven test have well-defined criteria and were considered as fully consent between the different examiners. In order to provide a fully comparable assessment of the WISC-R test, one psychologist rated performed answers for all the children.

### Confounders

Data on possible confounders such as maternal age (continuous variable) and education (university vs. non-university); maternal non-verbal intelligence (result of the TONI-3 as a continuous variable); older siblings (yes or no); exposure to passive tobacco smoking during pregnancy (yes or no); child’s gender, gestational age (≥37 vs. <37 weeks), birth weight (continuous variable, unit 100 g), Apgar score, blood mercury and lead level (cord and at the age of 5), breastfeeding (children breastfed for at least 6 months), and exposure to ethylmercury in later life (from the 29th day to the 6th month) were collected and included in the analysis.

### Statistical analysis

In the descriptive analysis, differences in the distribution of women and newborns’ parameters grouped by thimerosal vaccination status were tested using *χ* ^2^ (for nominal variables) and the Mann-Whitney and Kruskal-Wallis tests (for continuous variables).

The comparison of the test outcomes according to the exposure to the type of vaccine (TCVs vs. non-TCV-vaccinated group) was studied using multivariate linear models. The logistic models were used to assess risk of poorer developmental outcomes (MDI <85, Raven <75, IQ <110) as well. All variables from Table 1 showing a probable association with thimerosal vaccination status (*p* < 0.1) were included in statistical multivariable models. Blood lead level at the age of 5 was used as a confounder in models for 5-year-old and older children. Additionally, the child’s gender was added to all models as it is highly associated with developmental tests’ performance.

**Table 1:** Characteristics of the study subjects by neonatal and 6-month ethylmercury exposure

	Neonatal exposure to TCV					Thimerosal exposure up to 6 months						
	Non-exposed (*N* = 98)		Exposed (*N* = 220)		*p*	No exposure (*N* = 27)		From 25 to 50 μg (*N* = 117)		Above 50 μg (*N* = 174)		*p*
	*N*	%	*N*	%		*N*	%	*N*	%	*N*	%	
Maternal education level					0.536							
Elementary/ vocational school	11	11.2	17	7.7		1	3.7	4	3.4	23	13.2	0.009
High school	39	39.8	85	38.6		10	37.0	41	35.0	73	42.0	
University	48	49.0	118	53.6		16	59.3	72	61.5	78	44.8	
Maternal marital status	90	91.8	209	95.0	0.272	26	96.3	111	94.9	162	93.1	0.719
Poor economical status	4	4.1	20	9.1	0.118	0	–	7	6.0	17	9.8	0.146
Paternal education					0.985							
Elementary/ vocational school	12	13.0	27	13.4		2	7.7	7	6.7	30	18.4	0.006
High school	28	30.4	63	31.2		5	19.2	30	28.6	56	34.4	
University	52	56.5	112	55.4		19	73.1	68	64.8	77	47.2	
Passive tobacco smoking during pregnancy	47	48.0	72	32.7	0.010	14	51.9	31	26.5	74	42.5	0.006
Boys	49	50.0	119	54.1	0.500	16	59.3	65	55.6	87	50.0	0.508
Gestational age <37 weeks	4	4.1	7	3.2	0.743	3	11.1	8	6.8	0	–	<0.001
Older siblings	40	40.8	78	35.5	0.361	9	33.3	42	35.9	67	38.5	0.825
Breastfeeding up to 6 months	71	72.4	154	70.0	0.658	19	70.4	86	73.5	120	69.0	0.705
Passive tobacco smoking during 5 years	16	19.5	54	28.7	0.112	3	12.5	20	21.1	47	31.1	0.062
	Mean	SD	Mean	SD		Mean	SD	Mean	SD	Mean	SD	
Mother’s age at the 2nd trimester	27.5	3.84	27.6	3.52	0.907	28.3	3.94	28.0	3.31	27.1	3.73	0.073
Maternal non-verbal intelligence (TONI-3)	105.7	19.06	110.3	17.04	0.082	108.3	20.39	110.0	17.77	108.6	17.36	0.834
Birth weight [g]	3391.5	531.52	3412.4	469.47	0.726	3206.3	637.80	3391.0	518.85	3447.0	433.44	0.053
Head circumference at birth [cm]	33.9	1.65	33.8	1.44	0.962	33.3	1.88	33.9	1.52	33.9	1.42	0.404
Cord blood mercury level [μg/L]	0.9	0.44	1.0	0.68	0.300	1.0	0.57	1.1	0.76	1.0	0.56	0.570
Cord blood lead level [μg/dL]	1.7	0.91	1.3	0.49	<0.001	1.7	0.65	1.3	0.65	1.4	0.67	0.001
Blood mercury level [μg/L]	2.1	0.67	2.2	0.74	0.056	2.0	0.59	2.1	0.81	2.2	0.68	0.409
Blood lead level [μg/dL]	0.6	0.26	0.5	0.32	0.224	0.6	0.33	0.6	0.31	0.5	0.30	0.495

*SD* standard deviation, *p* significance level

Statistical analyses were performed using STATA software version 12.1.

## Results

The analyzed population consisted of 318 children: 52.8 % boys and 47.2 % girls. One child from that group (0.3 %) was absent during FTII and BSID-II test at the 12th month. In that group, retention rate during psychological tests in further years was 97.5 % at 2nd, 94.7 % at 3rd, 78.3 % at 5th, 60.7 % at 6th, 67.9 % at 7th, 55.0 % at 8th, and 64.8 % at 9th with reference to the initial cohort, respectively. Retention rates were comparable between studied groups.

In the analyzed group, all but one child were vaccinated against hepatitis B in the neonatal period, 220 (69.2 %) of them with TCVs. Ninety-eight (30.8 %) children were vaccinated with thimerosal-free Engerix B, 162 (50.9 %) with Euvax B, and 58 (18.2 %) with Hepavax-Gene, the latter two containing 25 μg thimerosal per dose. Most of the children (302, 95.0 %) were vaccinated within 1 day after birth and an additional nine (totally 97.8 %) within 1 week.

Exposure to TCVs up to the age of 6 months was connected with further doses of hepatitis B and DTPw/DTPa vaccines administered according to the Polish immunization schedule. The children were vaccinated with DTPw vaccine with thimerosal (25 μg/dose) manufactured in Poland and thimerosal-free vaccines (DTPa), such as Infanrix IPV+Hib, Infanrix Hexa, Tripacel, and Tetracoq. All the analyzed vaccines (thimerosal-containing and thimerosal-free) contained aluminum compounds as adjuvant.

**Fig. 1:**
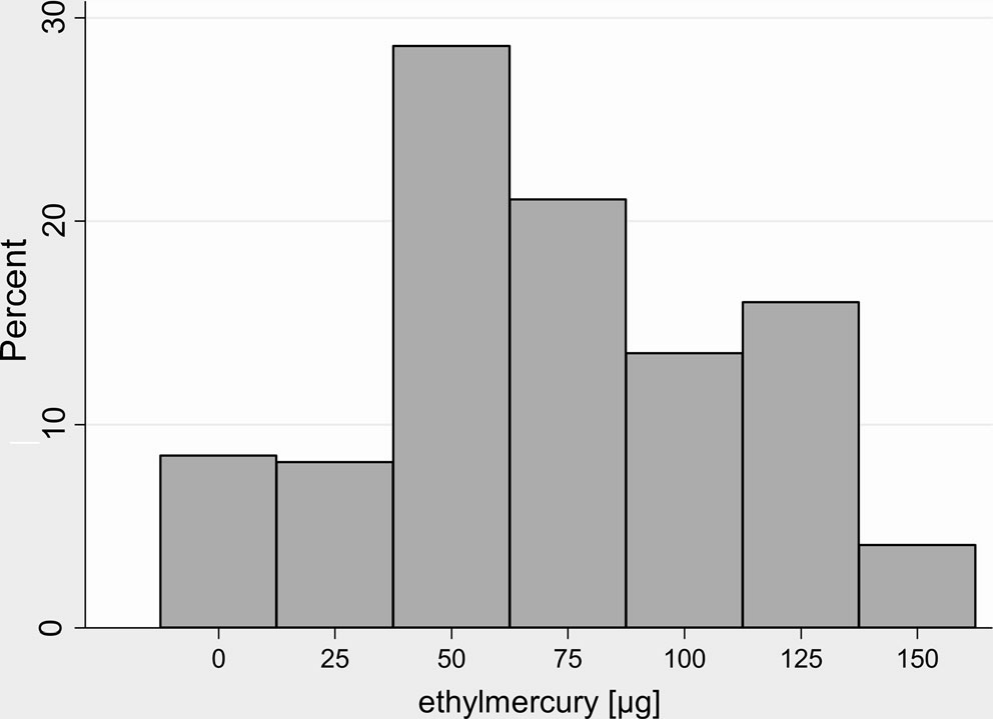
Distribution of ethylmercury doses in the 6-month period

Between the 29th day and the 6th month of life, 86.2 % of children were vaccinated with TCVs. The children immunized with ethylmercury-containing vaccines in the neonatal period were more frequently vaccinated with ethylmercury later in their life compared to those not immunized with TCVs (92.3 vs. 72.4 %, *p* < 0.001). Figure 1 presents the distribution of exposure to TCVs in the period of the first 6 months of life.

Neonatal exposure to TCVs was derived from hepatitis B vaccine only which was mainly administrated in the first 24 h after delivery. It was dependent on the decision of the Ministry of Health, who decided about the kind of vaccine (cheaper or more expensive) secured for all children in Poland at the same period of time. In the group of children examined, those born in 2001 were vaccinated almost only (89.2 %) with thimerosal-free vaccines, while those born in 2002–2004 with TCV (96.8 %) in the neonatal period. This vaccination was independent of parents’ decision. No significant differences were found for maternal characteristics such as age, education, and the TONI-3 scores, between the children exposed and non-exposed to TCVs in the neonatal period. Passive tobacco smoking during pregnancy was less common among the mothers of children from the neonatal TCV group (32.7 vs. 48.0 %, *p* = 0.010). On the other hand, further exposure to TCVs depended partly on the Ministry of Health decision and partly on family socioeconomic status that can be seen in significant differences in maternal and paternal education levels (Table 1). Also, prematurely born children were less often vaccinated with TCV.

As for child characteristics, only the lead level in cord blood was significantly lower in the TCV-exposed group than in the non-exposed one (1.3 vs. 1.7 μg/dL, *p* < 0.001, for exposure in the neonatal period and 1.3, 1.4, and 1.7 μg/dL in the 6-month period) (Table 1).

### The outcomes of cognitive development

**Fig. 2:**
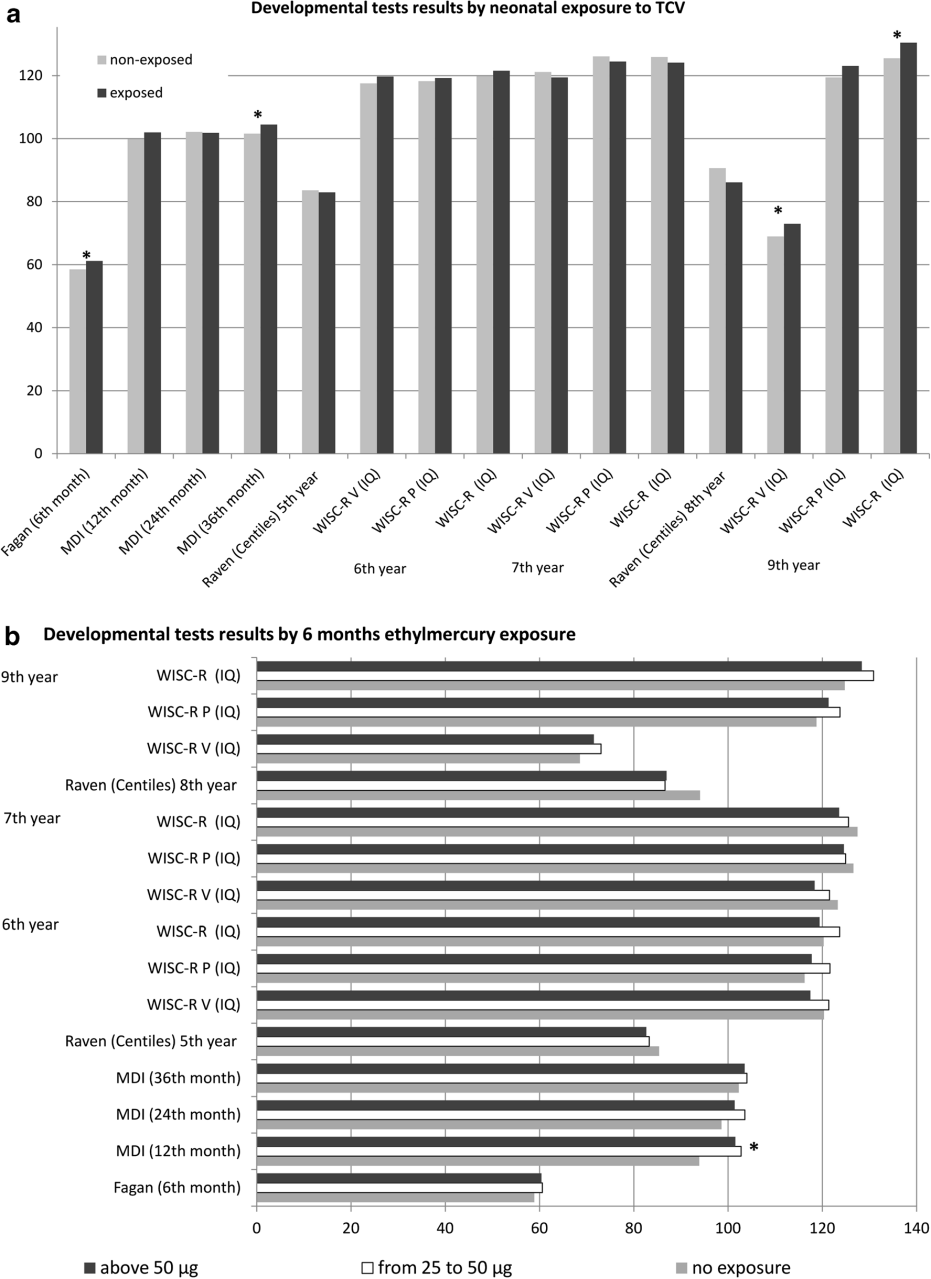
Average development test scores in the ethylmercury-exposed and non-exposed groups in the ** a) **neonatal ** b)** 6 month period. *p < 0.05. *MDI* Mental Development Index of BSID-II, *WISC-R V* WISC-R verbal scale, *WISC-R P* WISC-R performance scale

The results of psychological tests verifying children’s cognitive development were mostly on the same level in groups exposed and non-exposed to TCVs in both neonatal and 6-month periods, which were presented in Fig. 2a, b. The significant differences among exposures in the neonatal period were observed in the Fagan (58.5 vs 61.2, *p* = 0.002), the MDI at the 36th month (101.6 vs 104.6, *p* = 0.024), and the WISC-R at the 9th year according to verbal (69.0 vs 73.0, *p* = 0.003) and total (125.4 vs 130.4, *p* = 0.005) IQ levels, which were higher in the TCV-exposed groups. After standardization to child’s gender, mother’s higher education, prenatal ETS, breastfeeding up to 6 months, Hg in cord blood, as well as the exposure to thimerosal from the 29th day up to the 6th month, only the MDI of the BSID-II (*β* = 4.10, *p* = 0.016) and the WISC-R (*β* = 3.96, *p* = 0.040 for verbal IQ, and *β* = 4.73, *p* = 0.049 for overall IQ) differences remained significant (Table 2). All developmental tests performed from the 5th to 9th year were additionally standardized to blood mercury level which was measured when children were 5 years old. Significant differences in the MDI levels observed at the age of 12 months connected with thimerosal exposure during 6 months after birth were observed only in univariate analysis (93.9, 102.8, and 101.4, *p* = 0.003). After standardization to the above mentioned variables, they lost their significance (*β* = −0.09, *p* = 0.825) (Table 2).

**Table 2:** The association between ethylmercury exposure and developmental test scores up to 9 years of age

Test	Age	Neonatal exposure^a^			6-month exposure^b^		
		Beta	*p*	95 % CI	Beta	*p*	95 % CI
FTII	6th month	1.38	0.248	−0.97–3.72	0.14	0.632	−0.43–0.70
MDI of BSID-II	12th month of life	2.09	0.220	−1.26–5.44	−0.09	0.825	−0.90–0.71
MDI of BSID-II	24th month of life	−2.00	0.337	−6.08–2.09	−0.48	0.343	−1.46–0.51
MDI of BSID-II	36th month of life	4.10	0.016	0.76–7.43	0.78	0.060	−0.31–1.58
Raven (centiles)	5th year of life	−0.29	0.937	−7.50–6.92	−0.77	0.619	−2.45–0.92
WISC-R	6th year of life						
Verbal IQ^c^		2.32	0.399	−3.12–7.76	−0.62	0.337	−2.01–0.77
Non-verbal IQ^c^		3.31	0.279	−2.72–9.35	−0.70	0.374	−2.24–0.85
IQ^c^		2.98	0.256	−2.19–8.15	−0.73	0.273	−2.05–0.59
WISC-R	7th year of life						
Verbal IQ^c^		−1.66	0.513	−6.65–3.34	−1.12	0.265	−1.88–0.52
Non-verbal IQ^c^		−0.72	0.802	−6.41–4.96	0.43	0.534	−0.94–1.80
IQ^c^		−1.33	0.560	−5.87–3.19	−0.14	0.805	−1.23–0.96
Raven (centiles)	8th year of life	−3.40	0.325	−10.22-3.42	−0.35	0.691	−2.09–1.39
WISC-R	9th year of life						
Verbal IQ^c^		3.96	0.040	0.18–7.75	0.02	0.963	−0.99–1.04
Non-verbal IQ^c^		3.20	0.214	−1.87–8.27	−0.71	0.285	−2.02–0.60
IQ^c^		4.73	0.049	0.03–9.44	−0.40	0.527	−1.64–0.84

^a^Standardized to child’s gender, mother’s higher education, prenatal ETS, breastfeeding up to 6 months, Hg in cord blood, as well as exposure to thimerosal from the 29th day up to the 6th month

^b^Standardized to child’s gender, mother’s higher education, prenatal ETS, breastfeeding up to 6 months, and dHg in cord blood; exposure to ethylmercury as continuous variable formulated as number of TCV doses

^c^Additionally standardized to blood Hg level in 5-year-old children

**Table 3:** The risk of delay in cognitive development according to ethylmercury exposure

Test	Age	Neonatal exposure^a^			6-month exposure^b^		
		OR	*p*	95 % CI	OR	*p*	95 % CI
MDI of BSID-II	12th month of life	0.58	0.238	0.24–1.42	0.93	0.597	0.71–1.22
MDI of BSID-II	24th month of life	1.04	0.928	0.44–1.47	0.98	0.873	0.76–1.26
MDI of BSID-II	36th month of life	0.56	0.341	0.17–1.84	0.80	0.242	0.55–1.16
Raven (centiles)	5th year of life	0.93	0.835	0.47–1.84	0.99	0.923	0.82–1.20
WISC-R	6th year of life						
Verbal IQ^c^		0.75	0.457	0.35–1.59	1.15	0.216	0.92–1.44
Non-verbal IQ^c^		0.91	0.825	0.40–2.06	0.97	0.968	0.79–1.28
IQ^c^		1.16	0.736	0.47–2.90	0.97	0.812	0.75–1.26
WISC-R	7th year of life						
Verbal IQ^c^		0.79	0.576	0.35–1.78	1.04	0.717	0.83–1.32
Non-verbal IQ^c^		0.71	0.482	0.28–1.83	0.74	0.049	0.55–1.00
IQ^c^		0.52	0.222	0.18–1.49	0.81	0.231	0.57–1.14
Raven (centiles)^c^	8th year of life	2.69	0.224	0.55–13.19	1.07	0.609	0.83–1.37
WISC-R	9th year of life						
Verbal IQ^c^		0.45	0.076	0.19–1.09	1.00	0.984	0.77–1.30
Non-verbal IQ^c^		1.04	0.952	0.28–3.81	0.84	0.378	0.58–1.23
IQ^c^		0.42	0.248	0.09–1.84	0.76	0.247	0.47–1.21

^a^Standardized to the child’s gender, mother’s higher education, prenatal ETS, breastfeeding up to 6 months, as well as exposure to thimerosal from the 29th day up to the 6th month

^b^Standardized to the child’s gender, mother’s higher education, prenatal ETS, and breastfeeding up to 6 months; exposure to ethylmercury as continuous variable formulated as number of TCV doses

^c^Additionally standardized to blood Hg level in 5-year-old children

### Risk of poorer outcomes in cognitive development

According to assumed criteria, poorer outcomes in MDI results were present in 7.3 % of children at the 1st year, 9.0 % at the 2nd year, and 4.3 % at the 3rd year of life. Poorer outcomes of the Raven test were present in 20.9 % of children tested at the 5th year of life and 16.6 % of those tested at the 8th year of life.

WISC-R IQ results less than 110 were observed for 14.5–22.3 % of 6-year-old, 8.3–17.1 % of 7-year-old, and 3.9–14.1 % of 9-year-old children. No significant differences were observed in the proportion of children with poorer cognitive test results caused by neonatal TCV exposure in the case of all studied tests, but Raven in the 8th year. Similarly, we did not observe any differences in the proportion of children getting poorer results according to exposure to TCVs in the 6-month period. Multivariable analysis of risk of obtained poorer cognitive results from the 1st to 9th year of life did not show that TCV exposure may affect the studied results (Table 3).

## Discussion

We did not find any negative correlation between neonatal and early infancy TCV exposure and mental development in children up to 9 years of age. Our results are consistent with the observations of other authors who did not confirm the influence of TCVs on mental development [1, 8, 21]. On the other hand, some other reports indicated both beneficial and adverse effects of TCVs on children’s development [14]. This fact may result from the methodological differences of the epidemiological studies and the time elapsed between the exposure and the development assessment.

The advantage of our study is the long-term, 9-year survey of the cohort with methodological assessment of children’s development with an average 1-year interval between the administered tests. Differently from the other authors, we obtained the outcomes of children’s mental development after shorter and longer periods of time following early TCV exposure. Hence, we had the possibility to adjust the crude results to many important confounders that could influence development during the first years of life. The strength of the study is the consistency of the lack of association between early TCV exposure and mental development in each stage of life up to the 9th year of life after standardization to many important confounders. This consistency of outcomes during so many years of life gave us strong evidence that early TCV exposure has no harmful influence on later mental development.

The cohort in our study derived from the general population but was different from the other population studies. In our study, the breastfeeding rate at the 6th month was significantly higher compared to that of infants from the other cohorts [12]. On the other hand, there are studies related to thimerosal exposure and neurodevelopment, conducted in a cohort of exclusively breastfed infants [15]. High breastfeeding rate could be one of the reasons that there was no negative influence of early TCV vaccinations on mental development outcomes. This cannot be attributed solely to the protective role of breastfeeding because the development depends on multiple conditions that can eliminate the potential harmful effect of TCV exposure [19]. For example, in our cohort, girls had higher scores of mental development after TCV exposure compared to boys (not statistically significant). This fact could be derived from the protective effect of estrogen in girls and the negative influence of testosterone in boys [5].

The limitation of this analysis is the study cohort which may not be sufficiently representative of the general population. The main purpose of the Krakow Cohort Study was to assess whether the exposure of pregnant women to air pollutants affects future development of their children. To avoid biases from the other harmful factors, the enrollment covered only those pregnant women who were free from selected chronic diseases, smoking, and occupational exposure to chemicals. That made our cohort somewhat healthier than the entire population.

At first, our study was concentrated on TCV vaccinations in newborns, who greatly differ in their birth weight and maturity. These have an influence on ethylmercury toxicokinetics and toxicodynamics [18]. The newborns received different doses of ethylmercury per kilogram of body mass dependent on birth weight. Since immaturity is not a contraindication to hepatitis B vaccination, nearly all the newborns were vaccinated during the first days of life, independent of their birth weight and gestational age. In our cohort, the representation of newborns born at gestational age <37 weeks was relatively smaller compared to the general population. This special population of immature newborns with low and extremely low birth weight requires further studies.

The main strength of our study was a possibility to determine whether there is an association between early TCV exposure and mental delays of milder intensity. Most of the previous epidemiological studies were addressed to the links between ethylmercury exposure and more serious potential side effects in children. We revealed, using very precise mental development tests, that there is not even a subclinical harmful effect of early TCV vaccinations on mental development. The other strong point of our study was the active follow-up of pregnant mothers and their children. It gave us a possibility to control many important confounders potentially affecting children’s development. An adjustment to mercury exposure—prenatal (cord blood) and blood level at the 5th year of life—was the most important for that analysis. It is a very precise equivalent of mercury derived from the other sources, for example methylmercury from fish consumption that demonstrated harmful consequences on children’s neurodevelopment [13, 17]. Additionally, our analysis related to the neonatal period and included adjustment to post-neonatal TCV exposure up to the 6th month.

## Conclusions

We found no negative association between neonatal and early infancy TCV exposure and mental development in children up to 9th year of age. The results of our study should not be treated as a supportive argument for continuation of early TCV exposure if it is economically unnecessary. They may be interpreted as further evidence on the vaccines’ safety even if they still contain thimerosal.

## Abbreviations

BSID-II: Bayley Scales of Infant Development-Second Edition
DTP: Diphtheria-tetanus-pertussis
FTII: Fagan Test of Infant Intelligence
ETS: Environmental tobacco smoke
MDI: Mental Development Index of BSID-II
TONI-3: Test of Nonverbal Intelligence, Third Edition
TCVs: Thimerosal-containing vaccines
WISC-R: Wechsler Intelligence Scale for Children-Revised
